# Supplementary material for: Tissue-specific iron levels modulate lipid peroxidation and the FLASH radiotherapy effect
Source: Cell Death Dis. 2025 Sep 2;16(1):668. doi: 10.1038/s41419-025-07988-0 (PMC12405469; doi:10.1038/s41419-025-07988-0)
Supplement: Supplementary file 1 — Supplementary Figures [file 41419_2025_7988_MOESM1_ESM.pdf]

Fig. S1

(A)

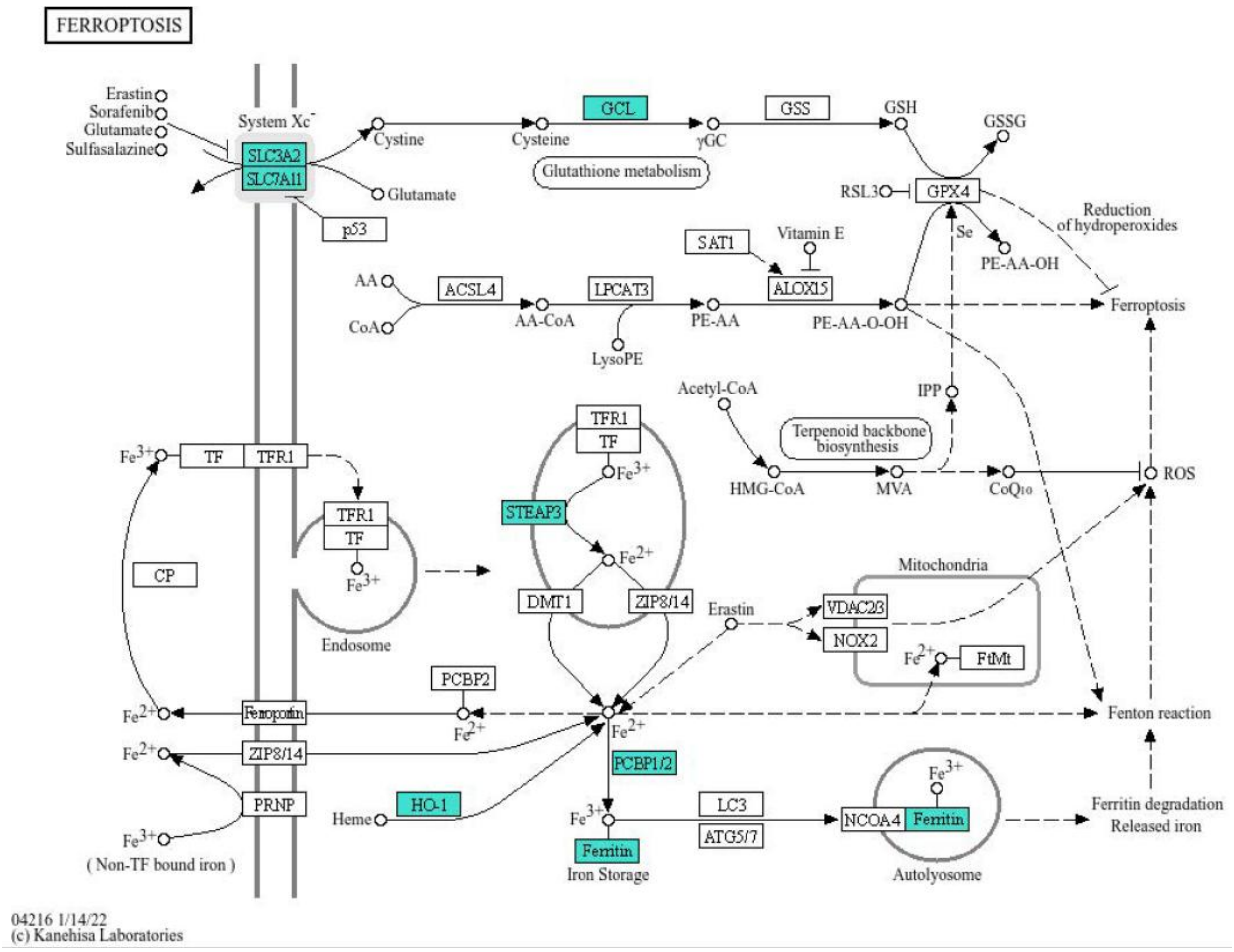

(B)

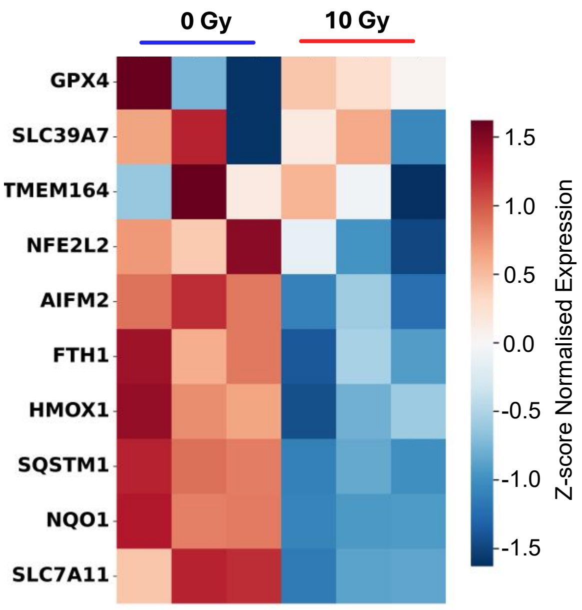

Fig. S1. Genes in the ferroptosis pathway were significantly altered by radiation. (A) The list of genes from RNA sequencing was analyzed by Enrichr and those involved in ferroptosis were visualized on the KEGG pathway map. (B) The heatmap generated by GSEA showed a downregulation of genes involved in ferroptosis suppression following radiation.

Fig. S2

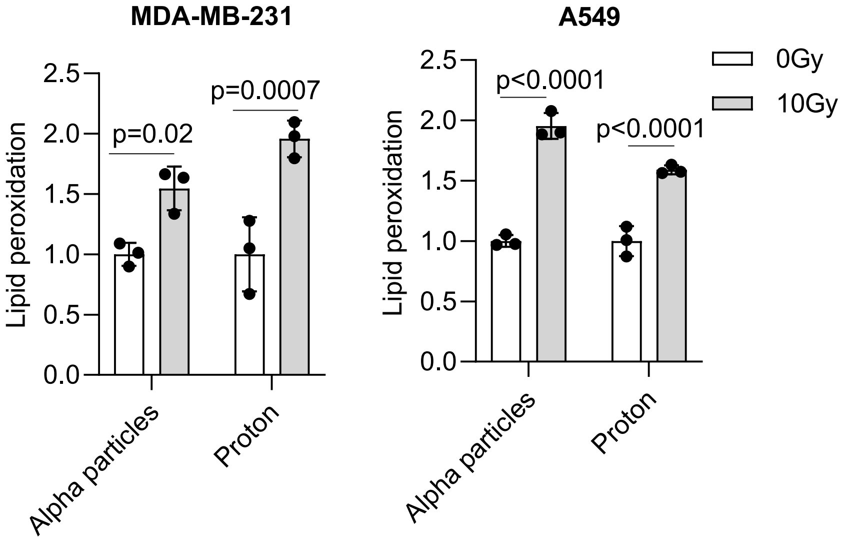

Fig. S2. Different radiation types similarly induced lipid peroxidation. MDA-MB-231 and A549 cells were treated with 10 Gy radiation by using alpha-particles or proton. Lipid peroxidation, which was measured 24 hours after RT, was significantly increased by both radiation types (n = 3). Error bars indicate standard deviation (SD). Statistical test was performed by Two-way ANOVA with Šídák's multiple comparisons test.

Fig. S3

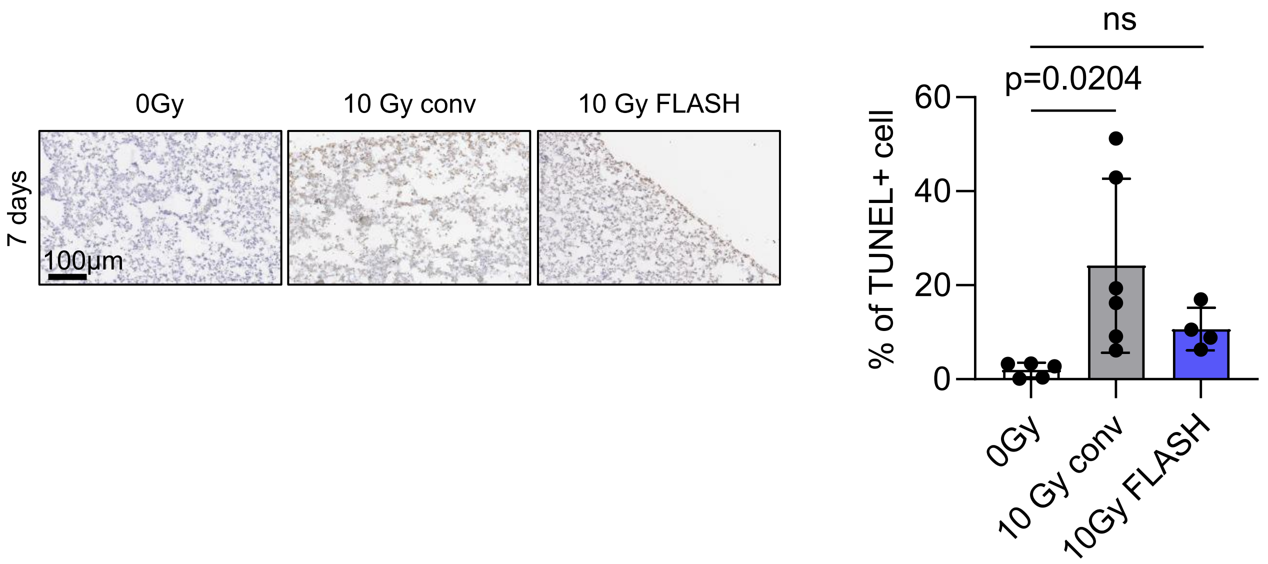

Fig. S3. Differential apoptosis by conventional and FLASH RT. Apoptosis in mouse lung tissues was assessed by TUNEL assay 7 days after treatment. While 10 Gy conventional RT significantly increased the population of TUNEL-positive cells, FLASH RT did not induce a comparable level of apoptotic damage.

Fig. S4

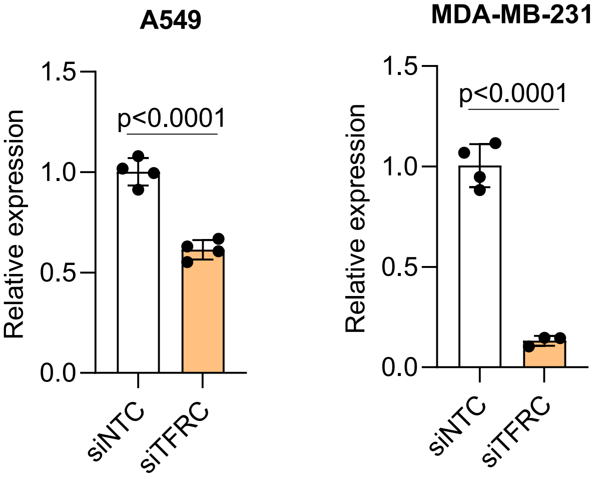

Fig. S4. Inhibition of TFRC expression. TFRC expression was inhibited by siRNA transfection in A549 and MDA-MB-231 cells. Significantly reduced expression after knock down was confirmed by qRT-PCR. (n = 3-4, student's *t*-test).

Fig. S5

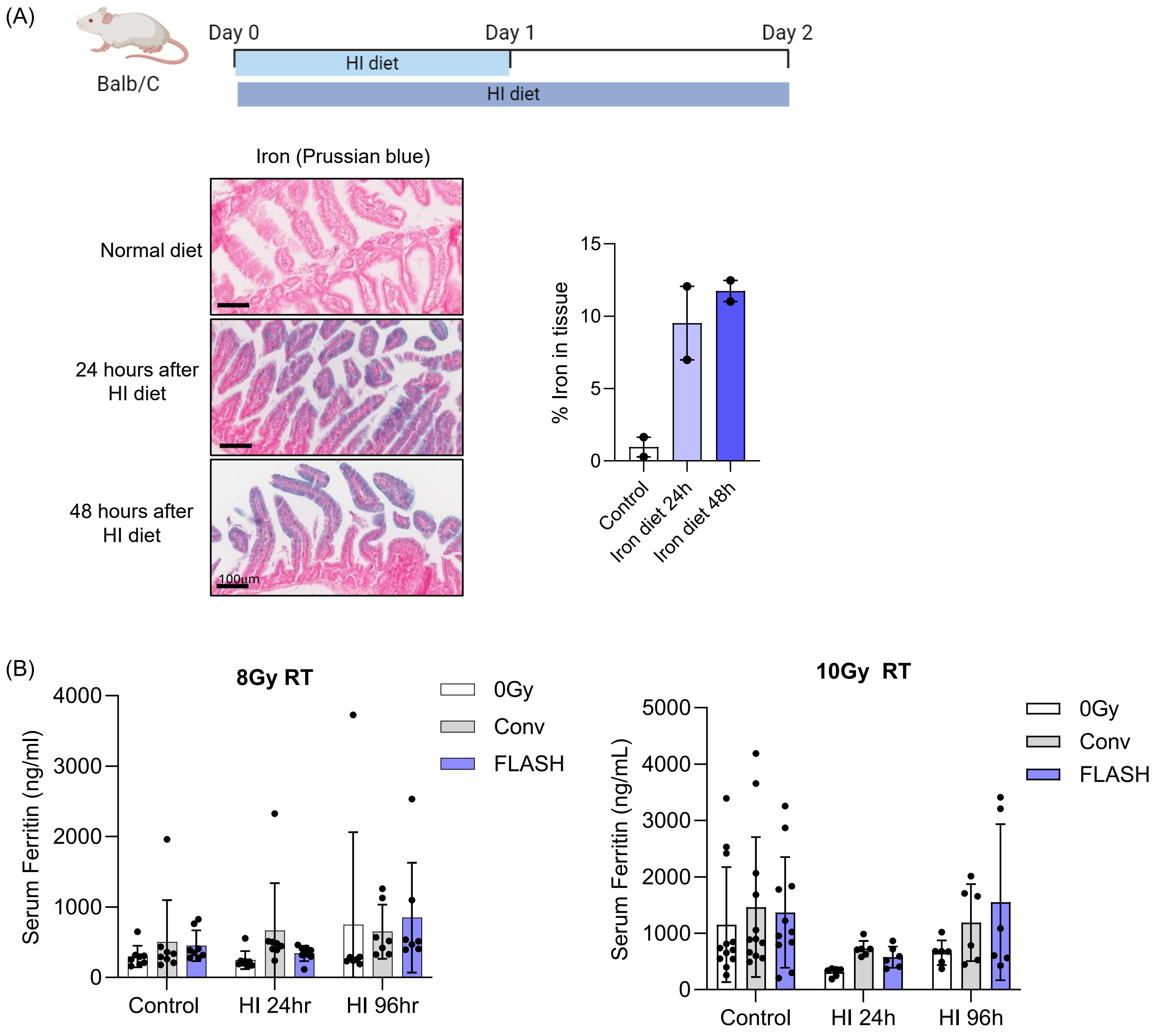

Fig. S5. FLASH spares intestine tissues and iron levels can be increased in the upper intestines by dietary supplementation. (A) Increasing dietary iron using an HI diet led to a rapid accumulation of iron in the upper intestines after 24 hours or 48 hours, as measured by using Prussian blue (n = 2). (B) Ferritin levels in the blood were not altered by a high-iron diet, indicating that it does not affect iron levels systemically (For 8 Gy, control diet; n = 8, HI 24-hourl n = 8, HI 96-hour; n=7, For 10 Gy, control diet; n = 12, HI 24-hour; n = 6, HI 96-hour; n = 6). Error bars indicate standard deviation (SD). Statistical tests were performed by One-way ANOVA with Tukey's multiple comparisons test (B).

Fig. S6

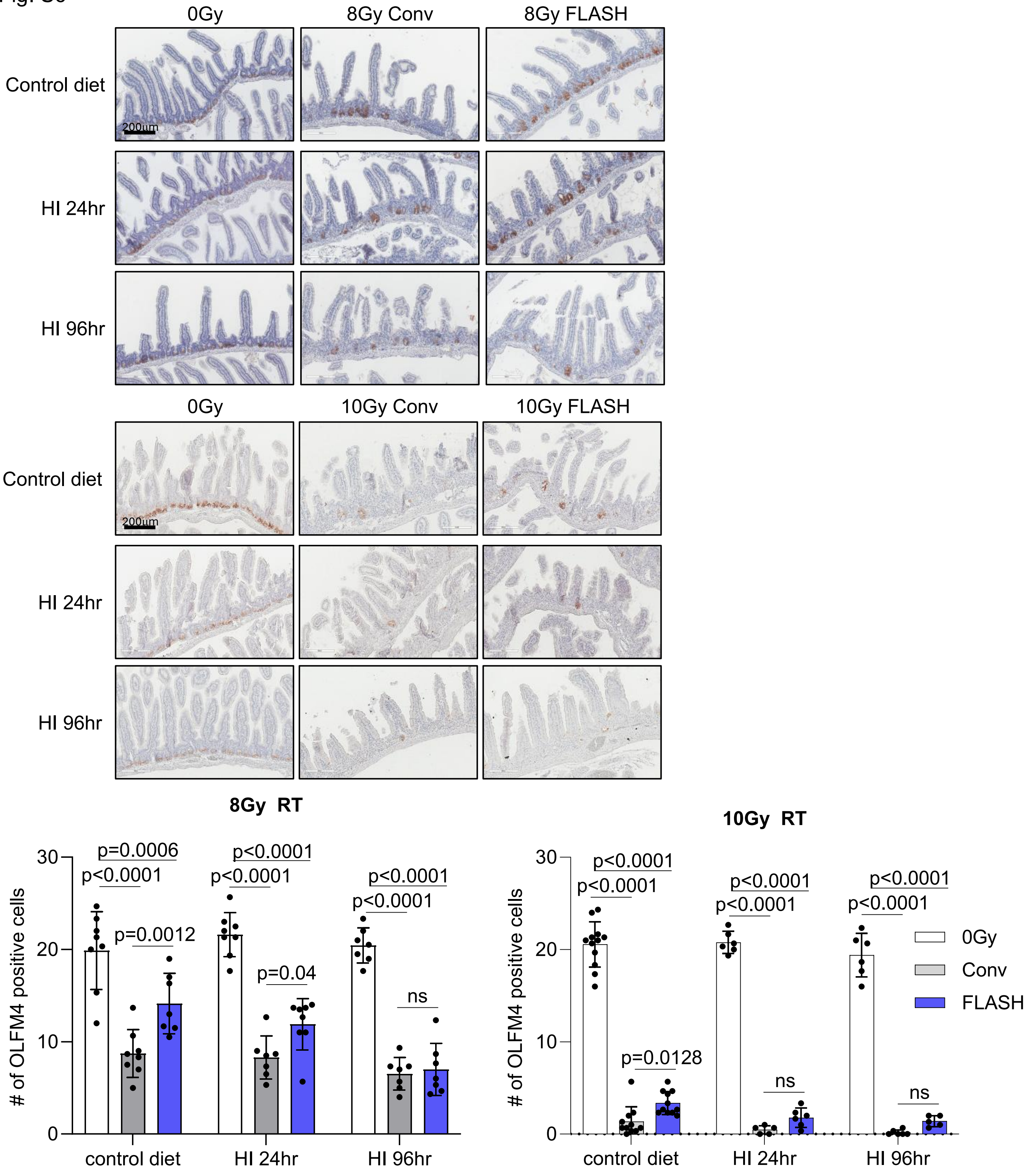

Fig. S6. Effect of iron diet on intestinal stem cell population. OLFM4 staining revealed that intestinal stem cells were significantly reduced following both 8 Gy and 10 Gy conventional RT. In contrast, FLASH RT resulted in less reduction of OLFM4-positive cells in the control diet group, indicating a protective effect. However, this protective effect was markedly diminished in mice on a high-iron diet.

Fig. S7

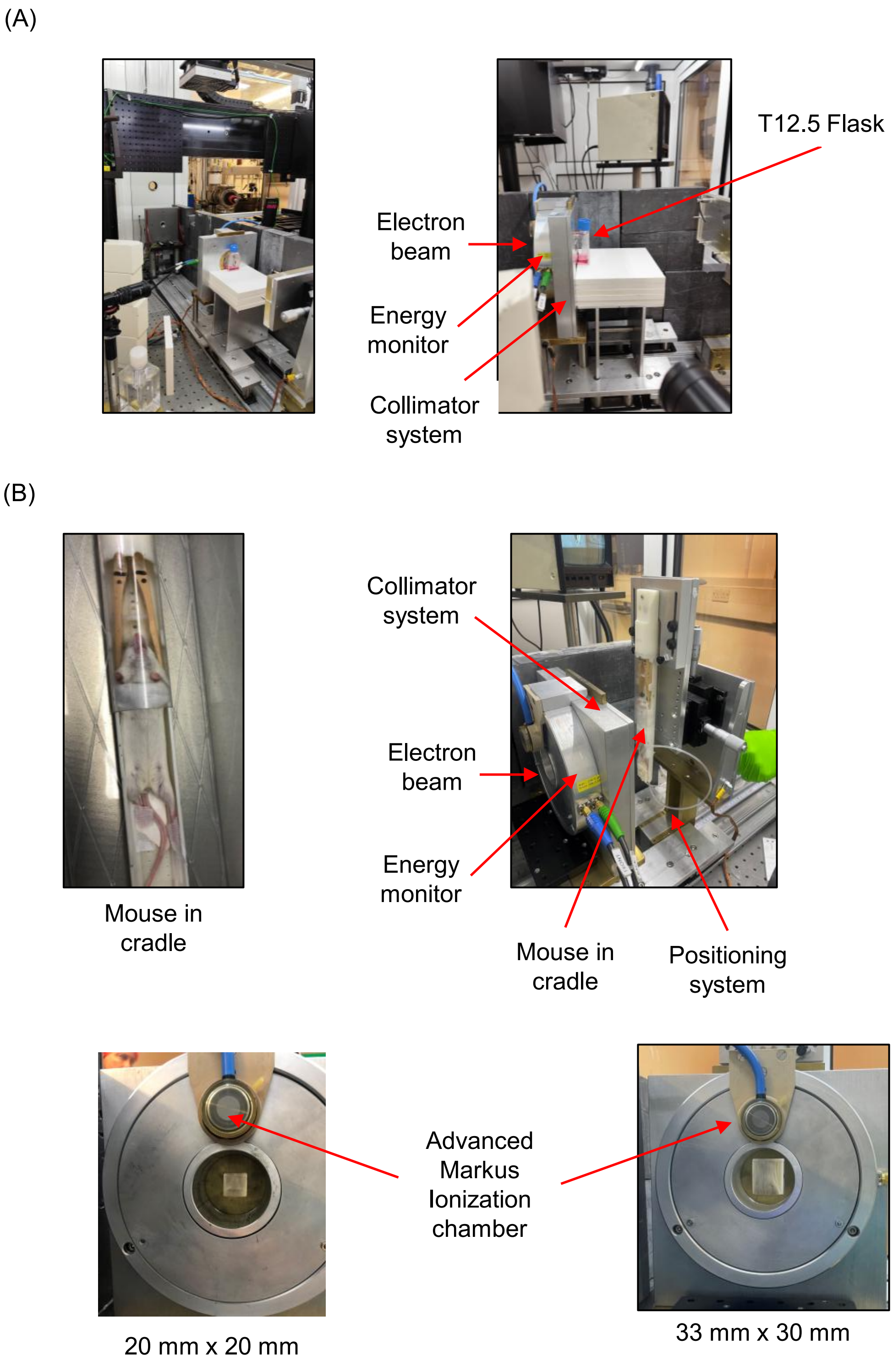

Fig. S7. FLASH RT is set up to treat both *in vitro* and *in vivo* models. (A) To perform *in vitro* studies T12.5 flasks or 35 mm cell culture dishes were irradiated in a vertical position in the central part of the beam. (B) The anesthetized mice were placed in an immobilization cradle and irradiated in an upright position. A 6 mm brass collimator was used to define the radiation field covering the whole abdominal area.

Fig. S8

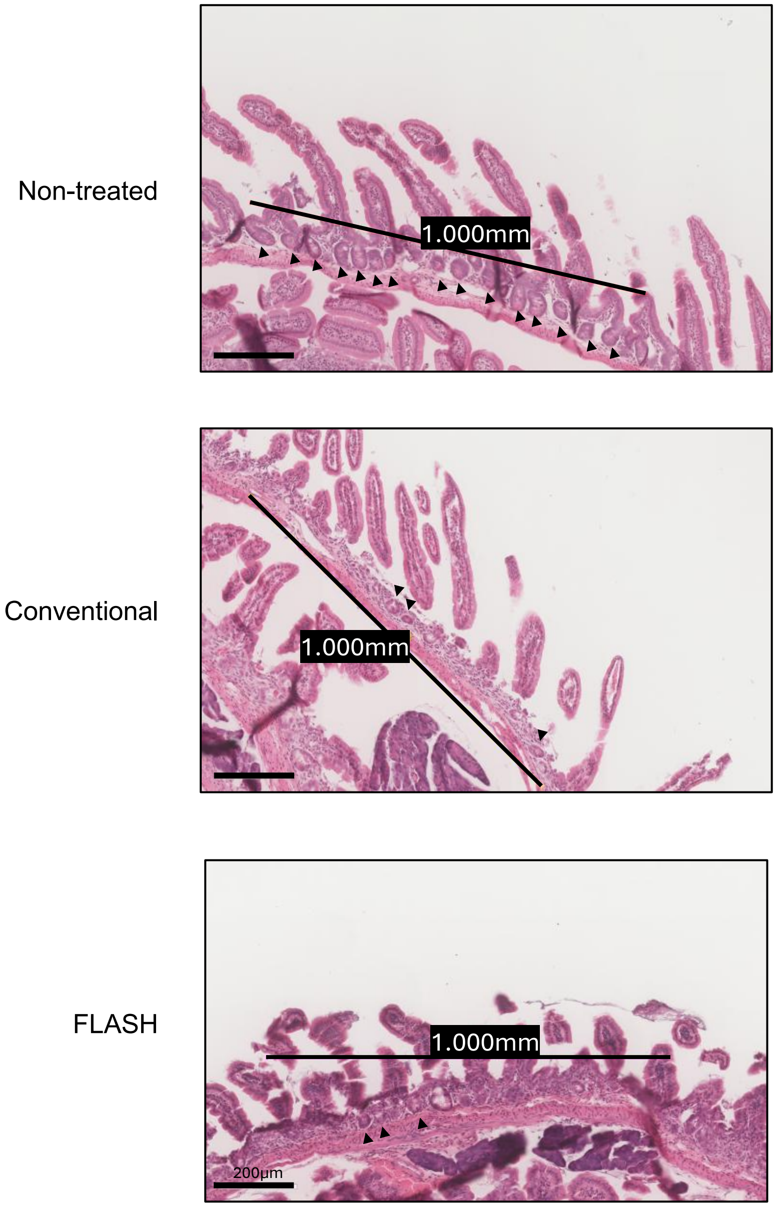

Fig. S8. The number of remaining crypts indicates radiation-induced damage. The number of remaining crypts in the upper intestines was quantified by selecting three representative fields, each 1 mm in size, per slide.
